# Microwave Crystallization of Lithium Aluminum Germanium Phosphate Solid-State Electrolyte

**DOI:** 10.3390/ma9070506

**Published:** 2016-06-23

**Authors:** Morsi M. Mahmoud, Yuantao Cui, Magnus Rohde, Carlos Ziebert, Guido Link, Hans Juergen Seifert

**Affiliations:** 1 Institute for Applied Materials—Applied Materials Physics (IAM-AWP), Karlsruhe Institute of Technology (KIT), Eggenstein-Leopoldshafen 76344, Germany; yuantao.cui@kit.edu (Y.C.); magnus.rohde@kit.edu (M.R.); carlos.ziebert@kit.edu (C.Z.); hans.seifert@kit.edu (H.J.S.); 2 Department of Fabrication Technology, Advanced Technology and New Materials Research Institute (ATNMRI), City of Scientific Research and Technological Application (SRTA), New Borg Al-Arab City, Alexandria 21934, Egypt; 3 Institute for Pulsed Power and Microwave Technology (IHM), Karlsruhe Institute of Technology (KIT), Eggenstein-Leopoldshafen 76344, Germany; guido.link@kit.edu

**Keywords:** microwaves, crystallization, LAGP, ionic conductivity, solid-state electrolyte, Li-ion batteries

## Abstract

Lithium aluminum germanium phosphate (LAGP) glass-ceramics are considered as promising solid-state electrolytes for Li-ion batteries. LAGP glass was prepared via the regular conventional melt-quenching method. Thermal, chemical analyses and X-ray diffraction (XRD) were performed to characterize the prepared glass. The crystallization of the prepared LAGP glass was done using conventional heating and high frequency microwave (MW) processing. Thirty GHz microwave (MW) processing setup were used to convert the prepared LAGP glass into glass-ceramics and compared with the conventionally crystallized LAGP glass-ceramics that were heat-treated in an electric conventional furnace. The ionic conductivities of the LAGP samples obtained from the two different routes were measured using impedance spectroscopy. These samples were also characterized using XRD and scanning electron microscopy (SEM). Microwave processing was successfully used to crystallize LAGP glass into glass-ceramic without the aid of susceptors. The MW treated sample showed higher total, grains and grain boundary ionic conductivities values, lower activation energy and relatively larger-grained microstructure with less porosity compared to the corresponding conventionally treated sample at the same optimized heat-treatment conditions. The enhanced total, grains and grain boundary ionic conductivities values along with the reduced activation energy that were observed in the MW treated sample was considered as an experimental evidence for the existence of the microwave effect in LAGP crystallization process. MW processing is a promising candidate technology for the production of solid-state electrolytes for Li-ion battery.

## 1. Introduction

The electrical energy storage systems have attracted a lot of attention in the past few decades because of the urgent need and the development of alternative energy sources. New generations of energy storage systems with large battery modules that are needed for the future electrical grid should have higher safety standards. The development of clean and highly efficient energy storage systems is becoming an even more urgent goal. As an electrochemical energy storage device, rechargeable lithium-ion batteries have been the dominant power sources for portable electronic devices due to their high energy density. They are also being pursued intensively for automotive and stationary storage applications.

Solid state electrolytes made of phosphate based ion conducting glass-ceramics are thermally more stable, non-flammable with higher melting points compared to the commercially used liquid and polymer based electrolytes [1,2]. They also have good mechanical stability that could stop the dendrite growth which causes serious problems in some liquid electrolyte based batteries [3].

Lithium aluminum germanium phosphate glass-ceramics (LAGP: Li_1+*x*_Al*_x_*Ge_2−*x*_(PO_4_)_3_) have a NaSICON (Sodium Super Ionic Conductor) structure. LAGP is consisting of two polyhedral [4,5,6], namely the GeO_6_-octahedra and the PO_4_-tetrahedra in which both polyhedra are linked to each other by their corners, forming a 3D-skeleton with bottlenecks. They are oriented along the c-axis through which the conducting Li-ions can pass [7]. Doping of the parent structure LiGe_2_(PO_4_)_3_ by Al^3+^ ions will introduce additional vacant lattice sites, which will enhance the Li-ion diffusion and consequently will lead to higher ionic conductivity with lower activation energy. Compared to liquid electrolytes with conductivity values in the order of 10^−2^ S/cm, the ionic conductivity of LAGP at room temperature is still relatively low and reaches maximum values in the order of 10^−4^ [8,9,10,11]. However, this is compensated at elevated temperatures [10,11,12], in which LAGP ionic conductivity value can reach or even exceed the liquid electrolyte corresponding value. A further unique and important issue for the transport processes in ceramic electrolytes is the effect of the microstructure [13,14] since grain boundaries as well as arrangement and size of the grains can be adjusted by a proper heat treatment, which will affect the overall ionic properties. LAGP solid electrolytes have been prepared via many different routes such as melt-quenching method [11,15,16], conventional powder sintering [17,18] and sol-gel methods [19,20]. LAGP synthesized from different routes had showed ionic conductivities values in the range of 10^−4^–10^−5^ S·cm^−1^ at room temperature. Several factors were found to influence the overall ionic transport process in LAGP such as the initial chemical composition, the developed crystal phases and impurities, the microstructure, the heat-treatment temperature and duration, and the used synthesis route and conditions [9,12,14,19,21,22,23,24]. Some selected LAGP ionic conductivities values prepared via different methods are shown in Table 1.

Microwave (MW) materials processing technology is a powerful tool to process materials. MW treated materials have shown several advantages when compared to the already used conventional materials processing techniques. In most cases, MW processing of materials is more efficient than conventional thermal processing due to the fact that MW energy is directly coupled with the material at the molecular level. The absorption of the microwave energy within the material depends on several factors such as the incident microwave frequency, the dielectric constant of the material, the dielectric loss of the material, and the distribution of the electric field within the material [25,26]. Many researchers [27,28,29,30,31,32,33,34,35,36,37,38,39,40,41,42,43,44,45,46,47,48] have reported enhanced kinetic rates, different reaction pathways, and/or different reaction products in several processes and materials using MW processing of materials when compared to conventional processing at the same or even at lower temperatures.

The possibility to use microwaves in the crystallization process of LAGP glass into ion-conducting glass-ceramics materials would be of great interest. Compared to conventional processing, MW processing of LAGP is expected to make the production of LAGP much faster with less associated costs and hence saving energy and money. In addition, MW processing has much less environmental impact as compared with conventional heating. It is also expected that MW processing could enhance the ionic conductivity of LAGP compared to the conventional method and hence a better battery performance could be achieved.

To the best of our knowledge, the use of high frequency MW in the processing of LAGP materials has not been investigated or reported yet. On the other hand, the use of microwave processing in the crystallization process of other glass systems into glass-ceramics materials with high mechanical properties such as lit hium disilicate (LS2) glass-ceramics has been successfully accomplished and reported by the author [49,50,51,52,53]. This accumulated experience and knowledge in the microwave processing of glass-ceramics and materials will be used to accomplish the goals of the current study.

## 2. Experimental Work

LAGP glasses were prepared using conventional melt-quenching method. Similar procedures were also employed in other studies [6,11]. The starting materials were Li_2_CO_3_ (Fluka, 99.0%, Buchs, Switzerland), Al_2_O_3_ (Sigma-Aldrich, 98.5%, St. Louis, MO, USA), P_2_O_5_ (Analar Normapur, 99.1%, Leuven, Belgium) and GeO_2_ (Alfa Aesar, 99.98%, Karlsruhe, Germany). Well-mixed starting materials powders were heated in a conventional electric furnace up to 1450 °C using a heating rate of 5 °C/min in an Al_2_O_3_ crucible and held at that temperature for 30 min. The melt was later quenched using a steel plate at room temperature and pressed with another steel plate to form a thin glass disc. The bulk glass pieces were then annealed at 450 °C for 1 h to remove the thermal stresses. The annealed glasses were finally crystallized and heat-treated using both 30 GHz MW processing and conventional heating at 800 °C for 6 h. These conditions have been adapted based on several conventional experimental trails and publications [9,14] in order to achieve the highest possible ionic conductivity to transform the glass into ion conducting glass-ceramic. For comparison, the same conditions have been used in the MW setup located at Institute for Pulsed Power and Microwave Technology (IHM), Karlsruhe Institute of Technology (KIT), Karlsruhe, Germany.

**Figure 1:**
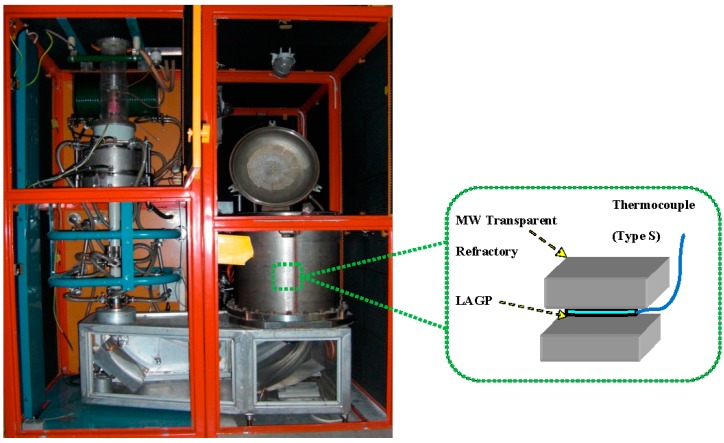
Thirty GHz microwave gyrotron system with a schematic of the lithium aluminum germanium phosphate (LAGP) glass crystallization setup.

The MW crystallization of the LAGP glass was done at the frequency of 30 GHz that was generated using a compact gyrotron system with a maximum power level of 15 kW [54]. The glass samples were placed in a MW transparent thermal insulation setup in order to reduce the heat loss from the heated glass samples to the environment and also to minimize the formation of cracks in the samples during MW processing. The MW experimental setup is shown in Figure 1. The temperature was controlled by a thermocouple (type S) that was in direct contact with the glass sample surface. On the other hand, a similar annealed LAGP glass sample was heat-treated conventionally in an electric furnace.

The chemical composition of the prepared glass was analyzed using inductively coupled plasma-optical emission spectrometry (ICP-OES, Optima 4300 DV, Perkin-Elmer, Wellesley, MA, USA). The oxygen content was measured using Carrier Gas Hot Extraction (CGHE, TC 600, Leco Co., Saint Joseph, MI, USA). The glass transition temperature (T_g_) and the crystallization temperature (T_x_) were determined using differential scanning calorimeter (DSC 404, Netzsch, Selb, Germany).

The microstructure of the prepared glass-ceramics samples was examined using scanning electron microscopy (SEM, Philips XL 30S, Amsterdam, The Netherlands) on fresh fractured surfaces. The developed crystalline phases in these samples were identified using X-ray diffraction (SEIFERT XRD 3003 PTS, Hamburg, Germany) with Cu Kα radiation.

The ionic conductivity of the glass-ceramics samples was measured using impedance spectroscopy (Sourcetronic 2826, Bremen, Germany) in the frequency range of 500 Hz–2 MHz. The principles of these measurements were explained elsewhere [55]. Gold electrodes were sputtered on both sides of the samples before they were fixed between two current collectors and the whole assembly was inserted into a furnace with two cables connected to the impedance analyzer outside. The ionic conductivity was measured from room temperature up to 250 °C.

## 3. Results and Discussion

The chemical composition of the prepared LAGP glass from the above mentioned starting materials is shown in Table 2 with the chemical formula Li_1.71_ Al_0.53_ Ge_1.36_ P_2.99_ O_11.9_. It was shown in one of the first studies on LAGP by Fu [11] that the nominal composition of Li_1+*x*_Al*_x_*Ge_2−*x*_(PO_4_)_3_ shows a broad maximum of the ionic conductivity as a function of *x*. These conductivity values vary slowly with a shallow maximum at *x* = 0.5 with σ_Ion_ = 4 × 10^−4^ S/cm. This current prepared LAGP glass composition had a slightly higher Li content than (*x* = 0.5).

**Figure 2:**
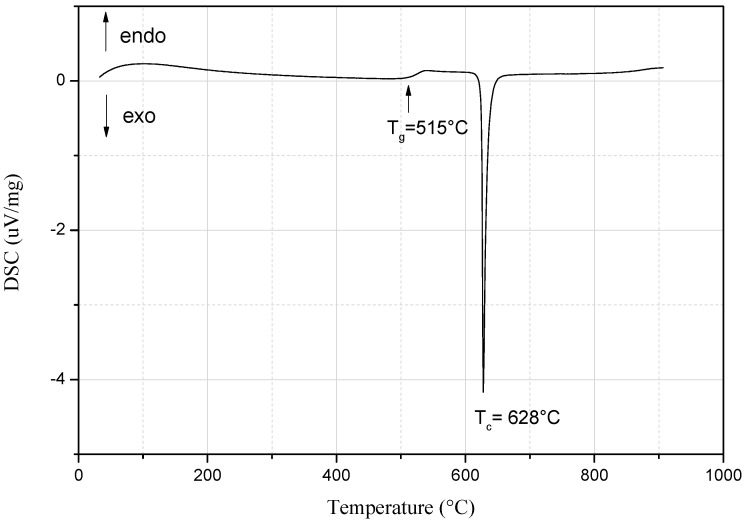
DSC of the prepared LAGP glass.

**Figure 3:**
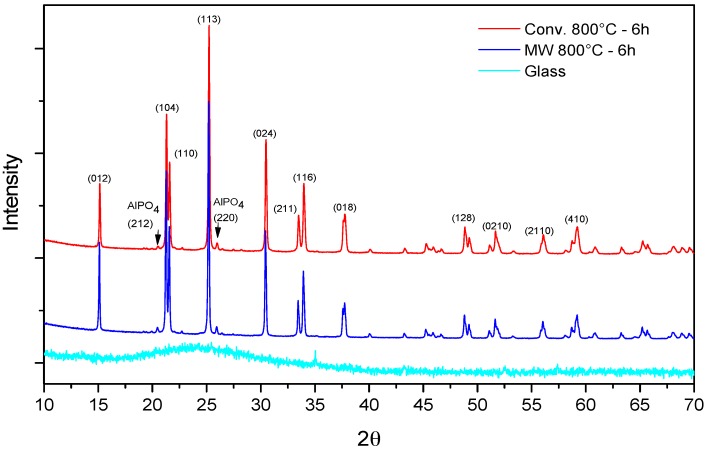
Comparison of XRD patterns of LAGP glass and glass-ceramics samples heat-treated at 800 °C for 6 h using 30 GHz microwave and conventional heating.

DSC measurement was used to determine the glass transition and the crystallization temperatures. The corresponding DSC trace of the prepared LAGP glass is shown in Figure 2. The glass transition was detected at 515 °C while the crystallization temperature was marked by the sharp exothermic crystallization peak at 628 °C. XRD measurement of the prepared LAGP glass powder revealed the amorphous structure formation (Figure 3) before subsequent heat-treatment via both MW and conventional heating in order to transform the LAGP glass into glass-ceramics materials.

Table 3 shows ionic conductivities values of some LAGP conventionally heat-treated samples at different temperatures and holding times. The annealed LAGP glass sample, i.e., below the crystallization temperature, showed non-ion-conducting behavior. This implies that the LAGP glassy phase is not a good Li ion conductor. On the other hand, LAGP sample conventionally heat-treated at or higher than 630 °C, i.e., around the crystallization temperature, had much higher ionic conductivity value than the annealed LAGP glass which was due to the formation of the major ion conducting LiGe_2_(PO_4_)_3_ crystal phase. During heat-treatment at 800 °C, the ionic conductivities increased gradually with increasing heat-treatment time. For the conventionally heat-treated sample at 800 °C for 6 h (optimized heat-treated sample), the highest ionic conductivity obtained value was 1.3 × 10^−4^ S·cm^−1^ at room temperature in which the ion-conducting LAGP crystal phase with its optimum microstructure along with some remaining porosity was developed. Other studies had showed a similar trend [9,14], where the non-ion-conducting LAGP amorphous glass can transform into a good ion-conducting glass-ceramic material via long and/or high temperature heat-treatment. This will enhance its crystallinity, which is needed for better ionic conductivity. On the other hand, the highest ionic conductivity value of the MW heat-treated LAGP sample at 800 °C for 6 h was 2.77 × 10^−4^ S·cm^−1^ at room temperature. Thus, the MW heat-treated sample showed higher ionic conductivity compared to the conventionally sample at the same optimized condition. Both ionic conductivities values of these optimized samples, either conventionally or MW processed, were a bit lower than the reported value when using similar melt-quenching route [11] or even using the sintering route [18], as shown in Table 1. This could be attributed to the difference in the initial chemical composition between the prepared LAGP glass and the others compared studied glass compositions, as shown in Table 2. This will consequently affect the developed crystal phases, as well as impurities, and hence affect the overall ionic conductivity. Alternatively, both samples showed higher values than the reported values using sol-gel route [19,20,24]. This could be attributed to the difference in the chemical composition as well as to the denser microstructure with fewer grain-boundary effects that melt-quenching route could produce compared to sol-gel route [20].

Furthermore, the 30 GHz MW crystallized LAGP sample at 800 °C for just 1 h showed a higher ionic conductivity value than the corresponding conventionally heat-treated sample for longer times with even more heat-treatments steps and condition at (550 °C/1 h + 630 °C/1 h + 800 °C/1 h). Also, this one hour MW ionic conductively value was relatively closer and only a bit lower than the optimized conventionally heat-treated sample at 800 °C for 6 h. Further discussions will be focused on the optimized LAGP samples with the highest ionic conductivity obtained via both methods.

XRD spectra of LAGP glass-ceramics samples that were obtained using both heating methods at these optimum conditions, 800 °C for 6 h, are shown in Figure 3. For both methods, the main ion-conducting phase was identified as LiGe_2_(PO_4_)_3_ (PDF 41-0034), which is the main parent ion-conducting phase with the same lattice structure. In addition, a trace of a minor phase in both samples was found and identified as AlPO_4_ (PDF 41-0044). XRD spectra showed that 30 GHZ MW processing was used successfully to crystallize LAGP glass into glass-ceramics materials with the development of the ion-conducting LAGP phase.

**Figure 4:**
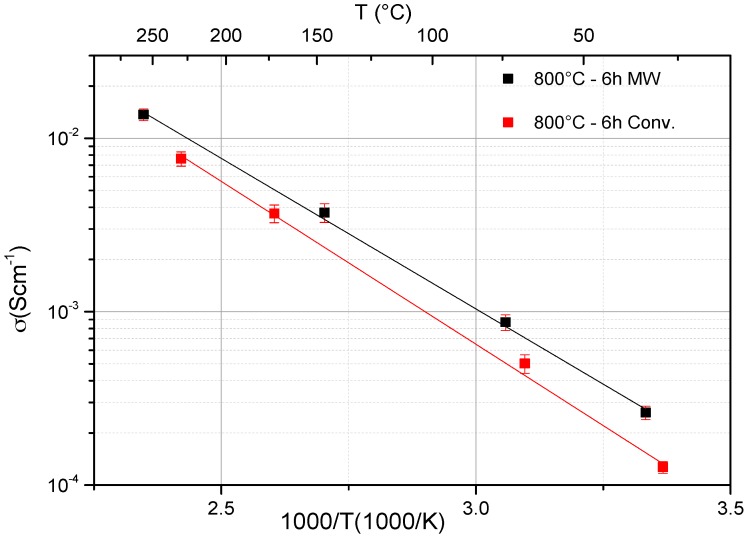
Ionic conductivity of LAGP glass-ceramics samples heat-treated at 800 °C for 6 h using 30 GHz microwave and conventional heating.

The ionic conductivities of the heat-treated LAGP glass-ceramics samples at 800 °C for 6 h, using 30 GHz microwave and conventional heating, are shown in Figure 4. The Arrhenius plots of both samples showed a small non-linearity at lower temperature, which was more noticeable in the conventionally heat-treated sample. This could be attributed to the existence of AlPO_4_ impurities [20,56], as revealed and confirmed from XRD patterns of both samples. The presence of AlPO_4_ in these LAGP samples will led to the formation of AlPO_4_:Li^+^ complex which exists only below 70 °C. This complex is a source of space charge and hence increases the activation energy for lithium ion transport [56].

**Figure 5:**
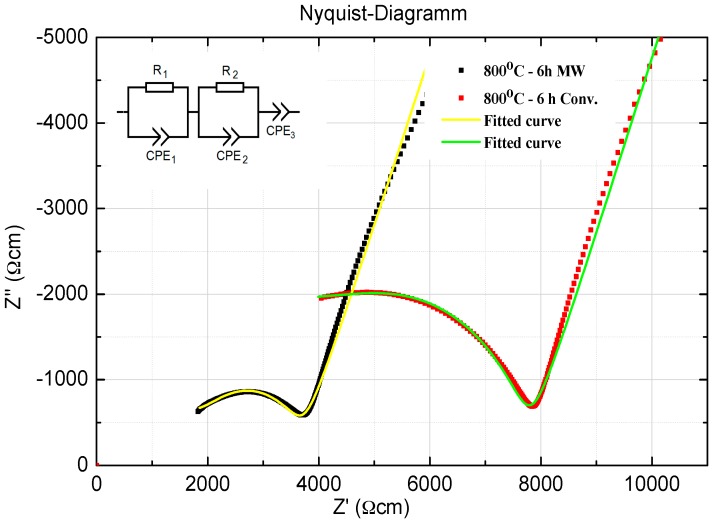
Impedance spectrum and equivalent circuit at room temperature (25 °C) of LAGP glass-ceramics samples heat-treated at 800 °C for 6 h using 30 GHz microwave and conventional heating.

From the impedance measurements of both samples, its corresponding Nyquist diagrams at room temperature were derived and shown in Figure 5 along with the standard equivalent circuit model (ECM) [57]. For both samples, the corresponding ECM consists of two distorted semicircles, which are represented by two parallel branches. Each composed of two resistances (R_1_, R_2_), two constant phase elements (CPE_1_, CPE_2_) and connected in series to a third constant phase element (CPE_3_). For both samples, R_1_ and R_2_ represent the grain and grain boundary resistance, respectively. According to Bouchet et al. [57], CPE_3_ is related to the almost vertical straight line that is observed at the lowest frequency (on the right side) and is corresponding to the capacitive blocking behavior of the electrodes. The diameter of the semicircle is equal to the resistance of the specimen at the particular temperature, which is further normalized with respect to the thickness and cross-sectional area of the specimen to obtain the total conductivity. On the other hand, it was not possible to perform the ECM analysis at the higher temperatures on the impedance plots since the semicircles representing the grains and grain boundary curves were stopped at 40 °C and no longer shown. Higher than this temperature, the grains contribution will shift to higher frequencies and it will be hardly observed and separated from the grain boundaries contribution in the used available measured frequencies range [15].

The total resistance of the samples is obtained from the right intercept of the semicircle with the real axis in the plots. It is concluded that the 30 GHz MW crystallized LAGP sample had higher total ionic conductivity at room temperature (2.77 × 10^−4^) compared to the conventionally treated LAGP sample (1.3 × 10^−4^). This trend was also observed at higher temperatures. Furthermore, the ionic conductivities of the grains and the grain boundaries were estimated using the “EIS Spectrum Analyzer” software. Table 4 shows total, grains and grain boundaries ionic conductivities of the optimized conventionally and MW heated-treated LAGP samples at room temperature. The total resistance of the sample is consisting of the grains and grain boundaries resistances. The high frequency semi-circle can be attributed to the LAGP grains conductivity while the low frequency to the grain boundaries contribution [15,57]. From Table 4, it is clear that both the grains and the grain-boundary ionic conductivities values of the MW optimized sample are significantly higher than their corresponding values of the conventionally optimized sample. This leads to the observed higher total ionic conductivity value of the MW treated sample compared to the corresponding conventionally treated sample at the same conditions. Since microwaves can interact with materials via different types of conductions and polarizations losses [25], it is believed that microwaves interfacial (grain boundary) polarization and relaxation have contributed significantly to the observed enhanced the grain boundary and the total ionic conductivities values in the MW treated sample as compared to the conventionally treated sample. These results can be considered as another experimental and strong evidence for the existence of the microwave effect in the LAGP crystallization process whereas comparable microwave effect was also reported in other glass systems [50,52].

On the other hand, the activation energy of the ion migration was evaluated from the slope of the straight lines in Figure 4 using the Arrhenius equation:

σ *= A·exp*(−*E_a_*/*k_B_T*) (1)

where *A* is the pre-exponential factor, *E_a_* is the activation energy for conduction, *k_B_* is Boltzmann’s constant and *T* is the absolute temperature [3,8]. The MW treated LAGP sample at 800 °C for 6 h exhibits an activation energy of 0.327 eV (±0.008), which is actually lower than the activation energy of the corresponding conventionally heat-treated sample which is 0.369 eV (±0.010). Thus, the 30 GHz MW crystallized LAGP sample with the optimum conditions developed a higher total ionic conductivity with lower activation energy and it had also lower grains and grain boundary resistance values compared to its corresponding conventionally treated LAGP sample. Activation energies values in the range of 0.3–0.4 eV were also reported by several researchers [15,21,56] for LAGP materials. The difference in the reported activation energies values could be attributed to the difference in the chemical composition, the microstructure, the developed crystal phases and impurities, the heat-treatment temperature and duration, and the used synthesis route and conditions. Table 5 summarizes the calculated activation energies for the ionic transport of both samples below and higher than 70 °C. Formation of AlPO4:Li^+^ complex below 70 °C is expected to increase the activation energy for lithium-ion transport in both samples and will act as a source of space charge. On other hand, the dissociation of this complex above 70 °C will eliminate the space-charge-mediated ionic transport and consequently the ionic transport will primarily occur through the LAGP grains and grain boundaries [56]. The MW optimized sample had lower activation energies values below or above 70 °C when compared to the optimized conventionally treated sample.

**Figure 6:**
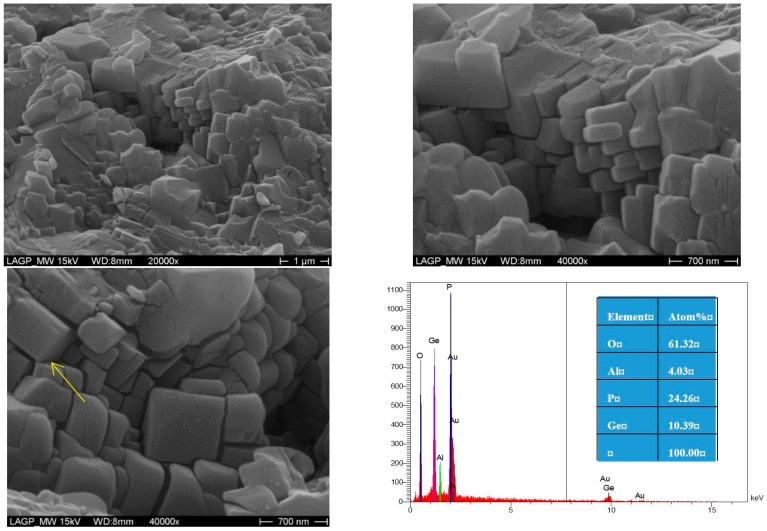
SEM micrographs with EDX data of LAGP glass-ceramics sample heat-treated at 800 °C for 6 h using 30 GHz microwave heating.

**Figure 7:**
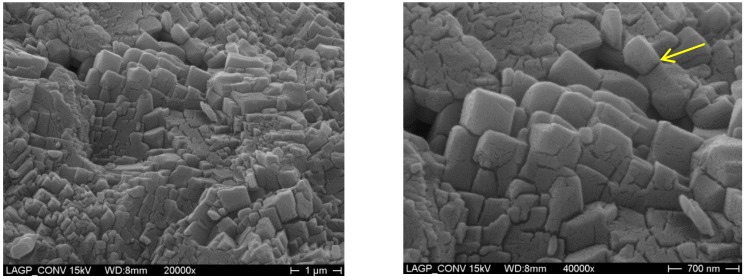

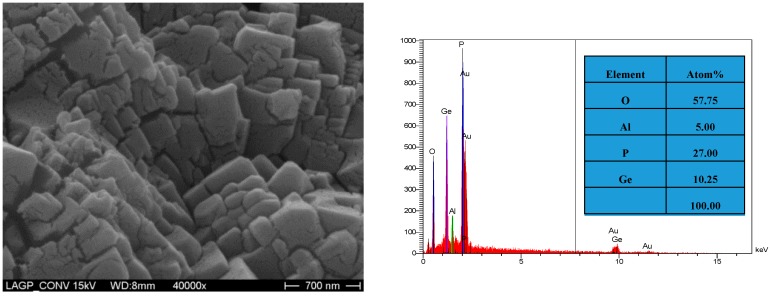
SEM micrographs with EDX data of LAGP glass-ceramics sample heat-treated at 800 °C for 6 h using conventional heating.

Figure 6 and Figure 7 show the SEM images on fresh fractured surface with EDX data of the LAGP samples crystallized at 800 °C for 6 h using MW and conventional heating, respectively. Both samples showed the typical microstructures of the major ion-conducting LAGP phase, as EDX data revealed, with some remaining porosity, which was actually affecting the overall ionic conductivity. The MW sample has relatively less porosity compared to the conventional sample, which affects and enhances the overall ionic conductivity. Furthermore, the conventionally optimized sample showed grain sizes in the range of 0.3–0.6 µm with the existence of few larger than 1 µm. On the other hand, the MW optimized sample showed grain sizes in the range of 0.3–0.9 µm with larger grains than 1 µm when compared to the conventionally treated sample. Thus, the MW optimized sample showed a relatively larger-grained microstructure with less porosity compared to the corresponding conventionally optimized sample which will eventually enhance both the grains and the total ionic conductivity values, as shown in Figure 4 and Figure 5 and in Table 4. Furthermore, the minor amorphous phase (denoted by arrows) that remains in the inter-granular interfaces will eventually affect the total conductivity of both samples. On the other hand, SEM pictures from both optimized samples do not show a significant difference in the amount of the remaining glassy phase between the grains. Thermal etching for the samples has been avoided while fresh fracture surfaces have been used in order to investigate the actual developed grains from both methods without any further grain-growth for comparison purpose. The observed features on the grains surfaces of both samples are due to the sputtered gold layer and not related to any intrinsic features in the developed grains.

In the future, it will be of great interest to investigate the effect of several factors such as: different MW frequencies, further optimization of MW heat-treatment conditions, and using different MW hybrid heating setups on both the ionic conductivity of LAGP solid-state electrolyte and on its developed microstructure.

## 4. Conclusions

30 GHz high frequency microwave processing was used successfully to crystallize lithium aluminum germanium phosphate glass composition into ion-conducting glass-ceramic materials without the aid of hybrid heating. The MW crystallized samples at the optimum heat-treatment conditions exhibited higher total ionic conductivity values over the measured temperature range with lower activation energy, relatively larger-grained microstructure with less porosity, as well as higher grains and grain boundaries conductivities when compared to the corresponding conventionally crystallized LAGP sample at the same conditions. The enhanced total, grains and grain boundary ionic conductivities values along with the reduced activation energy that occurred in the MW treated samples was considered as another experimental and strong evidence for the existence of the microwave effect in the LAGP crystallization process. Microwave processing technology is an attractive tool and offers new opportunities and promising alternatives in the production of solid-state electrolyte for Li-ion batteries. It might lead to faster and greener LAGP production process, reduce its production cost and save considerable amount of energy.

## Figures and Tables

**Table 1:** Selected lithium aluminum germanium phosphate (LAGP) ionic conductivities values obtained via different routes.

Route Type	Ionic Conductivity (S/cm) @ RT
Melt-quenching [11]	4 × 10^−^^4^
Conventional sintering [18]	3.99 × 10^−^^4^
Sol-gel method [20]	1.03 × 10^−^^4^
Flame spray [3]	2 × 10^−^^4^

**Table 2:** Chemical analysis and composition of the prepared LAGP glass.

Element	Li	Al	P	Ge	O
Weight %	2.85%	3.45%	22.20%	23.70%	44.40%
Atom %	9.43%	2.93%	16.45%	7.49%	63.70%
Chemical formula		Li_1.71_ Al_0.53_ Ge_1.36_ P_2.99_ O_11.9_			

**Table 3:** Ionic conductivities of some conventionally and MW heat-treated LAGP samples at different temperatures and holding times.

Sample	Total Ionic Conductivity (S/cm) @ RT
LAGP As annealed glass	NA *
LAGP Conv.(550 °C/1 h + 630 °C/1 h + 800 °C/1 h)	7.52 × 10^−^^5^
LAGP Conv. (800 °C/6 h)	1.3 × 10^−^^4^
LAGP 30 GHz MW (800 °C/1 h)	1.06 × 10^−^^4^
LAGP 30 GHz MW (800 °C/6 h)	2.77 × 10^−^^4^

* Too low to be measured.

**Table 4:** Total, grains and grain boundaries ionic conductivities of the optimized conventionally and MW heated-treated LAGP samples at room temperature.

Sample	Total Ionic Conductivity (S/cm) @ RT	Grains Ionic Conductivity (S/cm) @ RT	Grain Boundaries Ionic Conductivity (S/cm) @ RT
LAGP Conv. (800 °C/6 h)	1.3 × 10^−4^	3.06 × 10^−4^	2.25 × 10^−4^
LAGP 30 GHz MW (800 °C/6 h)	2.77 × 10^−4^	6.49 × 10^−4^	4.83 × 10^−4^

**Table 5:** Activation energies of optimized conventionally and MW heated-treated LAGP samples below and higher than 70 °C.

Sample	Below 70 °C	Above 70 °C	Overall Range
LAGP Conv. (800 °C/6 h)	0.44 eV	0.34 eV	0.37 eV ± 0.010
LAGP 30 GHz MW (800 °C/6 h)	0.38 eV	0.32 eV	0.33 eV ± 0.008
